# Limited evidence to assess the impact of primary health care system or service level attributes on health outcomes of Indigenous people with type 2 diabetes: a systematic review

**DOI:** 10.1186/s12913-015-0803-6

**Published:** 2015-04-11

**Authors:** Odette R Gibson, Leonie Segal

**Affiliations:** Health Economics and Social Policy Group, Division of Health Sciences, University of South Australia, Adelaide, 5001 Australia; Wardliparingga Aboriginal Research Unit, South Australian Health and Medical Research Institute, PO Box 11060, Adelaide, SA 5001 Australia

**Keywords:** Diabetes mellitus, primary health care, Indigenous people, Disease Management, Hospitalisation

## Abstract

**Background:**

To describe reported studies of the impact on HbA1C levels, diabetes-related hospitalisations, and other primary care health endpoints of initiatives aimed at improving the management of diabetes in Indigenous adult populations of Australia, Canada, New Zealand and the United States.

**Method:**

Systematic literature review using data sources of MEDLINE, Embase, the Cochrane Library, CINHAL and PsycInfo from January 1985 to March 2012. Inclusion criteria were a clearly described primary care intervention, model of care or service, delivered to Indigenous adults with type 2 diabetes reporting a program impact on at least one quantitative diabetes-related health outcome, and where results were reported separately for Indigenous persons. Joanna Briggs Institute critical appraisal tools were used to assess the study quality. PRISMA guidelines were used for reporting.

**Results:**

The search strategy retrieved 2714 articles. Of these, 13 studies met the review inclusion criteria. Three levels of primary care initiatives were identified: 1) addition of a single service component to the existing service, 2) system-level improvement processes to enhance the quality of diabetes care, 3) change in primary health funding to support better access to care. Initiatives included in the review were diverse and included comprehensive multi-disciplinary diabetes care, specific workforce development, systematic foot care and intensive individual hypertension management. Twelve studies reported HbA1C, of those one also reported hospitalisations and one reported the incidence of lower limb amputation. The methodological quality of the four comparable cohort and seven observational studies was good, and moderate for the two randomised control trials.

**Conclusions:**

The current literature provides an inadequate evidence base for making important policy and practice decisions in relation to primary care initiatives for Indigenous persons with type 2 diabetes. This reflects a very small number of published studies, the general reliance on intermediate health outcomes and the predominance of observational studies. Additional studies of the impacts of primary care need to consider carefully research design and the reporting of hospital outcomes or other primary end points. This is an important question for policy makers and further high quality research is needed to contribute to an evidence-base to inform decision making.

## Background

Type 2 diabetes (T2DM) is a chronic health condition that affects a significant and growing proportion of the global population. In 2013, an estimated 8.3% of world’s population aged 20 to 79 years had diabetes [1]. For the same period and age group, diabetes prevalence in Australia, Canada and the United States (US) was 10%, 10.2% and 10.9% respectively [1] and 8.1% in New Zealand (NZ) in 2011 [2]. By the year 2030 type 2 diabetes prevalence among the global population aged 20 to 79 years has been forecasted to increase by 69% in developing countries and 20% in developed countries [3]. These prevalence rates are average figures based on the total population and it is well established that prevalence of diabetes varies by population groups.

The evidence shows that the majority of the world’s Indigenous populations have been experiencing a more rapid increase in type 2 diabetes prevalence than their non-Indigenous counterparts [4]. This is the case in Australia, NZ, Canada and the US, all high income OECD countries [2] with well-established primary health care (PHC) systems, that for Indigenous populations are largely publicly funded. Whilst these populations have diverse cultures, languages and practices both within and between them which may warrant differences in interventions, there are also commonalities [5]. The Indigenous populations in these four countries have a shared history of colonisation that includes being displaced from their traditional lands which effectively removed their access to all known resources [6]. This has had devastating and lasting effects, that present in the current health disparities experienced by these Indigenous populations. These populations are all minority groups within their country and their population growth is faster than that of their non-Indigenous counter parts [5]. They all suffer poorer health outcomes and poorer social determinants of health than their non-Indigenous counterparts; and experience the on-set of type 2 diabetes at an earlier age and higher related morbidity and mortality [5].

Australia, NZ, Canada and the US are responding to this diabetes epidemic through policies that support mainstream and Indigenous specific health services to increase access to and quality of health care for Indigenous people. Primary health care services are recognised by the World Health Organisation as best positioned within the health system to detect and manage chronic diseases, including diabetes, from both an access and an economic perspective [7]. Chronic disease detection and management, although at different stages of implementation, are well embedded in the primary care setting in these high income countries [7].

It is necessary to manage diabetes well to prevent or slow the onset of related vascular damage, including cardiovascular [8] and kidney diseases [9]. The aim is to bring clinical risk factors to within the normal range to reduce the risk of diabetes-related vascular complications and mortality [10]. Large US and UK-based clinical trials, have demonstrated that raised HbA1c and blood pressure can be lowered, and poor blood lipid profiles improved, using structured treatment protocols [11]. However, despite evidence from randomised control trials (RCTs) of the benefits of intense diabetes management, care in the clinical practice setting still often departs from best practice, contributing to the poorer than expected health outcomes and higher health care costs [12,13]. Evidence linking better health outcomes with reduced hospital admission is also inconsistent, especially in real clinic settings [14,15].

In order to guide future policy to improve outcomes for persons with diabetes, especially for Indigenous populations—for whom prevalence of T2DM is highest and outcomes poorest—it is important to know the system level attributes that may contribute to the better management of type 2 diabetes in the primary care setting. The objective of this study was to assess the impact of PHC initiatives on health outcomes of Indigenous people in Australia, NZ, Canada and the US with T2DM, by systematically reviewing and synthesising peer-reviewed evidence.

## Methods

### Inclusion criteria

Participants were Indigenous adults with T2DM of Australia, NZ, Canada, or the US who have received diabetes management in a PHC setting. For the purpose of this review a PHC setting was defined as the provision of diabetes-related clinical care in the community, with the aim of preventing or reducing acute diabetes complications and/or diabetes-related vascular disease progression. A PHC intervention, model of care or service needed to be described and evaluated. This could include: i) the addition of a new service model incorporated into the local PHC service, for example, an outreach home visiting nurse providing individual hypertension management; ii) a system level quality improvement (QI) process to enhance the quality of diabetes care, for example, implementing evidence based diabetes care guidelines and support systems with related workforce development; or iii) a change in PHC funding/incentives, typically designed to increase access to care and/or support better quality care, for example, government reimbursement for completion of annual diabetes health check items. The study had to report a quantitative diabetes-related outcome; of HbA1c, diabetes-related hospitalisation and/or a diabetes-related health outcome. These outcome measures were chosen because HbA1c is the agreed indicator of individual glycemic control [16]. Unplanned diabetes-related hospital admissions are a well-accepted measure of how well a primary care service is performing [17]. Diabetes-related health outcomes such as lower limb amputation, renal failure (i.e. chronic renal disease and end-stage renal disease) and cardiovascular complications (i.e. coronary heart disease, heart failure and peripheral vascular disease) are also measures of access to, and quality of, PHC. In multi-ethnicity studies the results for Indigenous persons had to be reported separately. Study designs that were included for full quality assessment and data extraction were RCT, cluster randomised trials, pre and post cohort studies or multivariate analysis of cross sectional or longitudinal data.

### Search strategy

Medline, Embase, CINAHL and PsychINFO databases were searched. Search terms were identified using the map term search tool for Indigenous population groups of the four countries—Australia, NZ, Canada and the US—and T2DM. Search terms applied in Medline were Health Services OR indigenous people$ OR aborigin$ OR native born OR native people$ OR torres strait island$ OR American Indian$ OR native American$ OR Canadian Indian$ OR maori$ OR eskimo$ OR aleut$ OR nuit$ OR first nation OR pima OR cree OR cherokee OR American native$ OR American native continental ancestry group OR central American$ OR north American$ OR south American$ NOT Asian NOT brazil$ NOT latin$ AND exp *diabetes mellitus, experimental/or exp *diabetes mellitus, type 2/ OR (‘diabet$’ or ‘diabet$ complication$’) OR (non insulin dependent diabetes mellitus or diabetes mellitus type 2 or diabetes mellitus type ii or diabetes mellitus, non-insulin-dependent or dm2 or niddm or noninsulin dependent diabetes or noninsulin dependent diabetes mellitus or type 2 diabetes mellitus) OR (maturity onset diabetes mellitus or adult onset diabetes or adult onset diabetes mellitus or diabetes mellitus, maturity onset or diabetes, adult onset or maturity onset diabetes) NOT IDDM NOT insulin dependent diabetes mellitus NOT type 1 diabetes mellitus NOT child$ NOT maternal NOT pregnan$ NOT prevalence. Terms were searched for in the article title, abstract, substance word, subject heading and key words. The Cochrane library was searched for systematic review articles only. The search covered the time period from January 1985, to ensure evidence-based care model interventions were captured [18], to March 2012. A reference search was performed on included articles.

### Study selection

To determine eligibility for inclusion article titles were reviewed by author OG. Abstracts of eligible study titles were independently reviewed. Authors OG and LS reviewed 50 abstracts. OG and a colleague (DH) reviewed the remaining 88 abstracts. When agreement on eligibility for inclusion could not be reached by OG and DH, LS was consulted. Full manuscripts were reviewed by OG against the inclusion criteria and possible exclusions were discussed with LS to reach agreement. Templates developed by OG were used to record the assessment of each study against the eligibility criteria during the abstract review and the full article review.

### Assessment of methodological quality

Standardised critical appraisal tools, appropriate to the study design, were used to assess the methodological of articles that met the study inclusion criteria [19].

### Data collection

Data were extracted from included studies covering the characteristics of the study population, the PHC initiatives, the study design, quality and outcomes.

## Results

**Figure 1:**
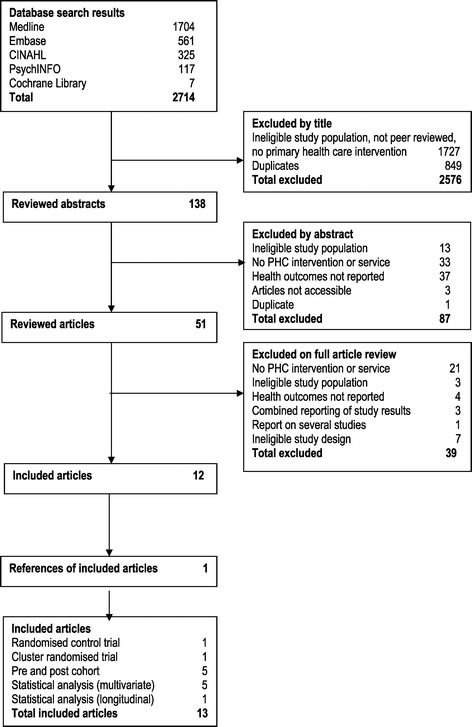
**Flowchart diagram.**

The electronic search identified 2714 articles, of which after initial screening, 51 full articles were reviewed. See Figure 1 for search outcomes. In total 13 articles met the inclusion criteria. Reasons for exclusion were, ineligible study population, no primary health care intervention implemented, did not report HbA1C/hospitalisation or health outcome, and results for Indigenous people were not report separately.

A brief description of each included study is as follows:

- ***Australian Coordinated Care Trials – late 1998 (Bailie et al. 2004)*** [20]
  Were conducted in two remote regions of the Northern Territory (NT); 1) Katherine West, which includes a township, several large communities and outstations and an Aboriginal population of approximately 3000; 2) the Tiwi Islands which has an Aboriginal population of approximately 1800 of a total of 2000. The CCT increased total primary care funds and facilitated the transfer of funds to local Indigenous health management boards for the purchase and delivery of health services. In addition, the trial increased the local Aboriginal health care workforce through access to certified training, and introduced clinical systems for providing systematic access to evidence-based chronic disease care. It commenced in late 1998 and ran for approximately two years. Results were presented up to three years post baseline data collection. Bailie and colleagues identified trends in diabetes processes of care and patient health outcomes before and after the trial at both locations.
- ***Improved Care Co-ordination Australia (Bailie et al. 2007)*** [21]
  A QI intervention was implemented between 2002 and 2005 in 12 PHC centres in the NT top end. The QI intervention included staff orientation and a conference to enhance understanding of the QI process (i.e. use of assessments and audits of clinical outcomes). The results informed the development of action plans, and facilitated sharing of progress across health centres. Bailie and colleagues explored system-related barriers and facilitators to improvements in outcomes of care by two means of assessment using their modified version of the Assessment of Chronic Illness Care Scale. They assessed 1) health system change and 2) diabetes quality of care by processes of care performed compared to clinical guideline recommendations and health outcomes by medical chart audit.
- ***Indian Health Service United States (Wilson et al. 2005; Roubideaux et al. 2008; Ramesh et al. 2008; Schraer et al. 2003)*** [22-25]
  The IHS is funded by the US Government to assist American Indian and Alaskan Native tribes establish and expand local PHC programs and diabetes teams. The diabetes initiatives of the IHS include increasing access to glucose monitoring supplies among American Indian and Alaskan Native communities, establishing a diabetes population register and operating a regional diabetes specialist referral centre. Wilson and colleagues [22] assessed the quality of diabetes care provided by the IHS for the period 1995–2001.
  **Integrated Diabetes Education Recognition Program (IDERP)** ranked IHS programs according to the comprehensiveness of PHC services provided. Level 3 ranking is the highest and is given to IHS services that deliver educational, clinical and public health programs. Only a level 3 ranking service is eligible to receive Medicare reimbursement for services performed. Roubideaux and colleagues [23] examined the association between the level of diabetes education program assigned by the IDERP (i.e. developmental and education and integrated combined) and the quality diabetes of care by the number of care items completed, such as eye examination, dental check, yearly education, and laboratory tests performed, across the 138 IHS sites.
  The **Special Diabetes Program for Indians** comprises multi-disciplinary diabetes teams that support the implementation of evidence based care guidelines and QI processes in IHS hospitals, regional health centres and tribal clinics. The program receives referrals from all IHS facilities and maintains a regional diabetes register. Ramesh and colleagues [24] assessed the quality of care delivered by the Special Diabetes Program for Indians by measuring the processes of evidence-based care achieved and patient health outcomes of 1394 participants, using a pre and post study design.
  The **High Risk Foot Program** devised a diabetic foot risk categorisation system and maintained a register of high-risk patients with diabetes for all IHS facilities. Specialist foot care outreach services were provided from the centre, which included training local health staff in foot care. Schraer and colleagues [25] compared the incidence of lower extremity amputation three years pre and three years post introduction of the High Risk Foot program.
- ***Care Plus – NZ (Kenealy et al. 2010)*** [26]
  An overall aim of Care Plus was to reduce health outcome inequalities between Maori and non-Maori. Launched as a QI initiative, NZ Government funding was available to PHC organisations to develop a wellness plan for all patients and employ nurse consultants to assist with diabetes management. Kenealy and colleagues conducted a pre and post cohort study to determine if the Care Plus initiative implemented by the Manaia PHO resulted in similar levels of glycemic control for Maori (n = 357) and non-Maori (n = 957) during July 2005 to August 2007.
- ***Get Checked – NZ (Smith et al. 2011)*** [27]
  The Get Checked program met patient costs of an annual diabetes review by reimbursing general practitioners (GPs) or practice nurses for completed diabetes care plans delivered in accordance with evidence-based care guidelines. Smith and colleagues conducted a longitudinal study, with a cohort of 295 people with T2DM in the Wellington Region of NZ, to investigate clinical outcomes over five annual visits during 2000 to 2006. A separate analysis was completed with Maori patients.
- ***Integrated diabetes specialist clinic – Australia (Simmons et al. 2003)*** [28]
  Simmons et al reported on the effectiveness of an integrated diabetes service in an Aboriginal Medical Service in rural Victoria. A weekly specialist diabetes clinic was integrated with the local PHC service. In between the weekly diabetes specialist visits, the local PHC team provided patient follow-up care. A retrospective audit of attendance at the clinic was linked to intermediate health outcomes of 47 patients with T2DM.
- ***Patient recall system to support evidence based guidelines for diabetes – Australia (McDermott et al. 2001)*** [29]
  Evidence-based guidelines for diabetes were provided to 21 PHC centres managed by Indigenous health workers in the Torres Strait Islands. In eight randomly identified intervention sites a paper-based diabetes recall system was established and staff training provided on the use of the system and basic diabetes care. McDermott and colleagues compared health indicators and hospitalisations collected at baseline (December 1998 to February 1999) and follow-up (March to April 2000) within and between the intervention and control sites. The aim was to determine if the diabetes recall system managed by local health workers improved the quality of diabetes care to patients and resulted in fewer diabetes-related hospitalisations.
- ***Diabetes Risk Evaluation And Macroalbuminuria 3 – Canada (Tobe et al. 2006)*** [30]
  DREAM 3 was the third study in response to the Battlefords Tribal Council to identify the prevalence of end stage renal failure in remote Saskatchewan communities. The study was an RCT focused on hypertension management. The intervention group of 50 participants received a nurse practitioner and local health worker managing and adjusting blood pressure control during regular home visits. The control group of 49 participants maintained usual GP care. Tobe and colleagues evaluated the effect of the community-based home visiting program on blood pressure control.
- ***Diabetes Outreach Van Enhancement program - Canada (Ralph-Campbell et al. 2006)*** [31]
  Promoting best practice guidelines and professional development of local health care professionals in diabetes care was the aim of the DOVE program. It was a regional-based specialist diabetes outreach team who delivered diabetes services in Northern Alberta rural health services. Ralph-Campbell and colleagues examined clinical differences between Aboriginal and non-Aboriginal Canadians who participated in the DOVE program, using a pre – post cohort design. There was no comparison group.
- ***Screening for Limb, I-Eye, Cardiovascular and Kidney (SLICK) complications – Canada (Virani et al. 2006)*** [32]
  A mobile outreach service provided by specialist staff screened for micro and macro-vascular complications of diabetes in Canadian First Nations people in Alberta. Its main aim was to support implementation of Canada’s clinical practice guidelines for diabetes through delivery of the outreach service and professional development of local health staff. Virani and colleagues evaluated the program.

The methodological quality of the four comparable cohort and seven observational studies was good and moderate for the two RCT (Table 1).

**Table 1:** **Methodological appraisal of studies**

**Comparable cohort/case control studies**									
**First author (Year)**	**Q1**	**Q2**	**Q3**	**Q4**	**Q5**	**Q6**	**Q7**	**Q8**	**Q9**
Kenealy et al. (2010) [26]	Y	N	Y	Y	Y	Y	N	Y	U
Smith et al. (2011) [27]	Y	Y	Y	Y	Y	Y	N/A	Y	Y
Schraer et al. (2003) [25]	Y	U	Y	Y^a^	Y	Y	Y	Y	Y
Ralph-Campbell et al. (2006) [31]	N	N	Y	Y	Y	N	Y	Y	Y
%	75	25	100	100	100	75	50	100	75
**Observational studies**									
**First author (Year)**	**Q1**	**Q2**	**Q3**	**Q4**	**Q5**	**Q6**	**Q7**	**Q8**	**Q9**
Bailie et al. (2004) [20]	Y	Y	Y^a^	Y	Y	Y	N	N	Y
Bailie et al. (2007) [21]	N	Y	Y	Y	Y	Y	N	N	Y
Roubideaux et al. (2008) [23]	N	Y	Y	Y	Y	U	N	Y	Y
Wilson et al. (2005) [22]	Y	Y	Y	Y	Y	Y	N	Y	Y
Simmons et al. (2003) [28]	N	Y	N	U	Y	Y	Y	N	Y
Ramesh et al. (2008) [24]	Y	Y	Y	Y	Y	Y	N/A	Y	Y
Virani et al. (2006) [32]	N	Y	N	Y	Y	N	N	N	Y
%	42.86	100	71.43	85.71	100	71.43	14.29	42.86	100
**Randomised control trial /Pseudo-randomised trial**									
**First author (Year)**	**Q1**	**Q2**	**Q3**	**Q4**	**Q5**	**Q6**	**Q7**	**Q8**	**Q9**
McDermott et al. (2001) [29]	U	N	N	N	U	Y	Y	Y	Y
Tobe et al. (2006) [30]	Y	N	N	Y	N	Y	Y	Y	Y
%	50	0	0	50	0	100	100	100	100

Note: Y – Yes, N – No, U- Unclear, N/A – Not applicable.

^a^Potential confounding factors were identified in qualitative research and not captured in statistical models.

Comparable cohort criteria: 1) Is the sample representative of patients in the cohort as a whole? 2) Are the patients at a similar point in the course of their condition/illness? 3) Has bias been minimised in relation to selection of cases and of controls? 4) Were confounding factors identified and strategies to deal with them stated? 5) Were outcomes assessed using objective criteria? 6) Was follow-up carried out over a sufficient time period? 7) Were the outcomes of people who withdrew described and included in the analysis? 8) Were outcomes measured in a reliable way? 9) Was appropriate statistical analysis used?

Observational criteria: 1) was the study based on a random or pseudo-random sample? 2) Were the criteria for inclusion in the sample clearly defined? 3) Were confounding factors identified and strategies to deal with them stated? 4) Were outcomes assessed using objective criteria? 5) If comparisons are being made, was there sufficient description of the groups? 6) Was follow up carried out over a sufficient time period? 7) Were the outcomes of people who withdrew described and included in the analysis? 8) Were outcomes measured in a reliable way? 9) Was appropriate statistical analysis used?

Randomised control trial criteria: 1) Was the assignment to treatment groups truly random? 2) Were participants blinded to treatment allocation? 3) Was allocation to treatment groups concealed from the allocator 4) Were the outcomes of people who withdrew described and included in the analysis 5) Were those assessing outcomes blind to the treatment allocation 6) were the control and treatment groups comparable at entry? 7) Were groups treated identically other than for the named interventions? 8) Were outcomes measured in the same way for all groups? 9) Were outcomes measured in a reliable way? 10) Was appropriate statistical analyses used?.

Four initiatives were located in Australia [20,21,28,29], three in Canada [30-32], two in NZ [26,27] and four in the US [22-25]. Ten initiatives were multifaceted with a focus on improving the quality and coordination of clinical care, increasing access to care and professionally developing the local workforce [20-26,28,29,32]. Three initiatives supported local health care providers to deliver optimum care [27,30,31]. Five of the 13 initiatives facilitated health system investment [20,22,23,26,27]. Six initiatives invested in building the capacity of the clinical system [21,24,25,28,29,32]. Two initiatives were new models of care [30,31]. Governance of the PHC services involved in the 13 primary care initiatives were community control health organisations (27%), government health departments (27%), shared government and community (36%), and private practice and community (10%). Eight initiatives were conducted onsite at the local PHC centre or general practice [20-23,26-29], four were visiting outreach services [24,25,31,32] and one initiative was a home-based care model [30]. For a summary of key study elements see Table 2. The source and level of program funding was not always clear. It would seem, however, that in most cases the initiative involved additional funding and additional resources.

**Table 2:** **Summary of key elements of the 13 primary health care initiatives**

**First author, year published, country**	**Intervention level**	**Governance**	**Setting(s)**	**Location**	**Delivery**	**Result reported**	**Method quality** ^**a**^	**Health outcome** ^**b**^
Bailie et al. 2004 [20]	HS	Shared	PHC	Remote	Clinic	HbA1c	7/9	Unchanged
Australia								
Bailie et al. 2007 [21]	CS	Shared	PHC	Remote	Clinic	HbA1c	6/9	Improved
Australia								
Roubideaux et al. 2008 [23]	HS	Shared	PHC, Hospitals	Urban & rural	Clinic	HbA1c	6/9	Unchanged
US								
Wilson et al. 2005 [22]	HS	Shared	PHC, Tribal Clinics, Hospitals	Remote & regional	Clinic	HbA1c	8/9	Improved
US								
Kenealy et al. 2010 [26]	HS	Private & CC	GPs	Urban & rural	Clinic	HbA1c	6/9	Improved
NZ								
Smith et al. 2011 [27]	HS	Govt.	GPs	Urban	Clinic	HbA1c	8/8	Unchanged
NZ								
Simmons et al. 2003 [28]	CS	CC	PHC	Rural	Clinic	HbA1c	5/9	Improved
Australia								
Schraer et.al. 2003 [25]	CS	Shared	Specialist referral service, PHC	Remote & regional	Out-reach	Amputation incidence	8/9	Improved
US								
Ramesh et al. 2008 [24]	CS	Shared	Specialist referral service, PHC	Remote & regional	Out-reach	HbA1c	8/8	Improved
US								
Virani et al. 2006 [32]	CS	CC	Mobile van	Remote & rural	Out-reach	HbA1c	4/9	Unchanged
Canada								
McDermott et al. 2001 [29]	CS	Govt.	PHC’s	Remote	Clinic	HbA1C	5/9	Unchanged
Australia						% hospitalised		Improved
						% hosp. episodes		Improved
Tobe et al. 2006 [30]	SP	CC	Community	Remote	Home visits	HbA1c	7/9	Unchanged
Canada								
Ralph-Campbell et al. 2006 [31]	SP	Govt.	PHCs	Regional centres in rural areas	Out-reach	HbA1c	6/9	Unchanged
Canada								

^a^Number of quality criteria met compared to total number of criteria.

^b^Outcome is reported as unchanged if the p value is insignificant, see results Table 3.

HS – health system; CS – clinical system; SP – service program; Shared – both government and community control; CC – community control; Govt. – government; PHC – primary health care service.

HbA1c levels were reported by 12 of the 13 studies. Schraer and colleagues [25] reported incidence rates of lower extremity amputation (Table 3). In addition to reporting HbA1c, McDermott et al. [29] reported the number of persons hospitalised for a diabetes-related reason and the number of diabetes-related admissions. A statistically significant improvement in HbA1c levels was reported by five of the 12 studies to report HbA1c, and seven studies reported no significant change pre – post cohort or compared with control or less-intensive management. McDermott et al. [29] reported a decrease in the proportion of persons hospitalised and episodes of diabetes-related hospitalisations among the group receiving the intervention. The specialist foot program appeared successful with a 59% reduction in the incidence of lower limb amputation between 1996 and 1998 compared to 1999 and 2000 [25].

**Table 3:** **Study results of health outcomes (HbA1c, diabetes–related hospitalisation, primary endpoints)**

**Initiative, study design (first author and date published)**	**Result reported**	**Baseline (or basic service level) [n]** ^**a**^	**Final visit or comparison group [n]** ^**a**^	**Difference**	**95% Confidence interval or P value**
CCT, pre – post cohort	HbA1c mean mmol/L% (CI)	9.0 (8.6, 9.4) [n = 137]	8.8 (8.3, 9.2) [n = 146]	−0.2	0.23 for trend
(Bailie et al. 2004) [20]					
Improved care coordination, cohort follow-up	HbA1c mean mmol/L% (CI)	9.3 (8.8, 9.8) [n = 295]	8.9 (8.6, 9.3) [n = 252]	−0.4	−0.7, –0.1
(Bailie et al. 2007) [21]					
IDERP – IHS, cross sectional	HbA1c mmol/L% (% patients <7.0)	not reported	not reported	OR = 1.1	0.8, 1.7
(Roubideaux 2008)^b^ [23]					
IHS, repeated cross section	HbA1c mean mmol/L% (SE)	8.9 (0.04) [n = 7110]	7.9 (0.03) [n = 15537]	−1.0	0.0001 (1995 vs 2001)
(Wilson et al. 2005) [22]					
Care Plus, open prospective cohort	HbA1c mean mmol/L% (CI)^c^	8.1 (8.0, 8.2) [n = 354]	7.2 (6.7, 7.5) [n = 3]	−0.9	<0.05 (based on CIs)
(Kenealy et al. 2010) [26]					
Get Checked, pre – post cohort	HbA1c mean mmol/L% (SD)	8.0 (1.6) [n = 298]	8.0 (1.6) [n = 298]	0	Not reported^d^
(Smith et al. 2011) [27]					
Integrated diabetes service, pre – post cohort	HbA1c mean mmol/L% (SD)	10.4 (2.2) [n = 30]	7.9 (1.9) [n = 30]	−2.5	<0.001
(Simmons et al. 2003) [28]					
Foot program – IHS, pre – post incidence study (Schraer et al. 2003) [25]	Amputation incidence (per 1000 person years)	16.4 (1342 person years)	6.8 (1628 person years)	−59%	0.021
Special diabetes program, repeated cross section	HbA1c mean mmol/L%	8.4 [n = 1394]	7.4 [n = 1839]	−1	<0.001
(Ramesh et al. 2008) [24]					
SLICK, pre – post cohort	HbA1c Mean mmol/L%	8.12 [n = 285]	8.01 [n = 285]	−0.11	0.176
(Virani et al. 2006) [32]					
Evidence based management of diabetes, cluster randomised trial	HbA1c mmol/L% (% <7)	not reported [n = 555]	IG = 22 CG = 20 [n = 678]	RR: 1.26^e^	0.219
(McDermott et al. 2001) [29]	Persons hospitalised for diabetes reasons (%)	IG = 20 CG = 22 [n = 555]	IG = 12 CG = 20 [n = 678]	IG = –8% CG = –2%	IG = 0.012 CG = 0.514^f^
	Diabetes hospital episodes (%)	IG =23 CG =30 [n = 555]	IG =19 CG =29 [n = 678]	IG = –4% CG = –1%	IG = 0.015 CG = 0.746^f^
Hypertension management, randomised unblinded control trial	HbA1c mean mmol/L% (SD)	IG = 7.9 (1.9)^g^ CG = 7.7 (1.8) [n = 99]	IG 7.8 (2.1) CG 7.7 (1.9) [n = 95]	IG = –0.1 (1.7) CG = –0.0 (1.3)	>0.05^h^
(Tobe et al. 2006) [30]					
DOVE, pre – post cohort	HbA1c mean mmol/L% (CI)	7.4 (7.0–7.8) [n = 94]	7.7 (7.4–7.9) [n = not reported]	+0.3	>0.05 (based on CIs)
(Ralph-Campbell et al. 2006) [31]					

Notes:

^a^Number of Indigenous participants.

^b^Roubideaux et al. [23] compared patient health outcomes of primary health care services awarded 1) service recognition of educational or integrated quality and 2) those that were in developmental stages of achieving an integrated service. Those in stage 2, more advanced stages of development, were 10% more likely to have a higher proportion of patients with a HbA1c < 7. This finding was not a statistically significant finding.

^c^HbA1c predicted from multivariate model.

^d^Smith et al. [27] report a net effect (measured from baseline to, at 5 years) of 0.03% increase in HbA1c for Maori and a 0.18% increase for European, both adjusted for age and gender.

^e^Interpreted as: Individuals at intervention sites are 26% more likely to have a HbA1c of <7 mmol% compared to those at control sites.

^f^P value for difference between intervention and control group not reported.

^g^Typographical error in Table 2 of original study manuscript reporting HbA1c level of intervention at baseline.

^h^Same result for comparison between groups over time and within group over time.

CCT – Coordinated Care Trial; CI – confidence interval; IDERP – Integrated Diabetes Education Recognition Program; IG – intervention group; IHS – Indian Health Service; mmol/L – millimoles per litre; SLICK – Screening for Limb, I-Eye, Cardiovascular and Kidney; DOVE – Diabetes Outreach Van Enhancement; CG – control group; RR – risk ratio.

## Discussion

The aim of the review was to assess the literature on the impact of primary health care initiatives for type 2 diabetes on health outcomes of Indigenous populations. We sought to do this by exploring consistency in findings of studies that related system or service level attributes of primary health care to improvements in objective diabetes health outcome measures. This would shed light on what might be contributing at a service or system level to what works to improve the quality of primary care in Indigenous populations. Since the introduction of evidence-based care guidelines, 13 studies published from 2001 that met the inclusion criteria were found. All four countries had published two or more studies. Six of 13 interventions reported lowered HbA1c levels or reduced diabetes-related hospitalisations or the incidence of amputation. All of the interventions were complex and varied across all of the elements of interest. This is to be expected, given the differences in health policies for Indigenous people across the four countries and the epidemiology of diabetes.

All six interventions that achieved an improvement in health outcome were multi-faceted in nature, delivering more than one component. For example, the Australian study that implemented evidence-based guidelines for diabetes in Torres Strait Islander health centres [29] included the establishment of population registers, recall and reminder systems, and patient care plans. The model was also supported by the necessary administrative and clinical resources to implement the guidelines and training and development of the workforce. Interventions to improve diabetes management in the primary care setting that address a combination of elements have been found to be more successful in achieving health outcomes than those that only focus on one element, such as a training program for health professionals [33]. Ten of 13 studies in this review were multifaceted interventions and six of these achieved an improvement in an objective health outcome measure.

Interventions included in this review were designed to introduce a health system, clinical system or service level program. It was not clear that the ‘level of intervention’ was predictive of a particular outcome. Of the five studies that implemented system level interventions, two achieved a reduction in HbA1c. One of these, an IHS initiative [22], involved the transfer of funds to Indigenous health organisations or tribal groups, whilst NZ Care Plus [26] financed local primary care providers to employ four practice nurses and provide all clients with a wellness plan and free health consultations [27]. Whilst the NZ initiative was unique amongst the five health system interventions, the IHS approach that involved the transfer of funds to Indigenous organisations was the focus of two other interventions [20,23]. A fifth study that offered people with diabetes a free annual health check [27] did not achieve a reduction in HbA1c.

Generally it was found that a strong primary health care system within a country is associated with lower rates of all-cause, premature and chronic disease-related mortality within the population [34]. The role of primary health care within the overall health system can be strengthened by re-orienting investment to include more primary care services and/or more practitioners per capita [35]. Mainstream policy reforms may increase service use by those population groups that have a greater ability to maximise the opportunity while the more vulnerable harder to reach groups may be less likely to benefit [36]. The Indigenous specific health system interventions that did not achieve an improved health outcome identified a range of impediments. These included implementation challenges pertaining to remoteness, the quality of communications infrastructure and transportation, the willingness of patients to participate in more comprehensive care, governance arrangements [20] and recruiting and retaining staff [20,23]. Bailie et al. [20] recommended that policy makers and researchers work to overcome challenges for sustained improvement in health outcomes in the Indigenous community controlled health sector.

Clinical system interventions were implemented by six of the thirteen reviewed studies. They each introduced a systematic evidence-based approach to diabetes management in local PHC settings and also professionally developed their workforce. For example, the Special Diabetes Program for Indians [24] comprised multi-disciplinary diabetes outreach teams that implemented clinical governance initiatives. This involved the introduction of evidence-based care guidelines, quality assurance programs, patient registers, and supporting and professionally developing the workforce. The program also received referrals from all IHS facilities and maintained a regional diabetes register. Factors of successful implementation of clinical governance have shown to include professional leadership, local relevancy, the ability for staff to review their own performance and readily accessible information on health care practice [37,38]. Four of the six clinical system interventions in this review achieved an improvement in the objective health outcome measure of interest.

There was a range of governance structures of the primary health care services that participated in the interventions. The majority of interventions were located in a primary health care service that had shared governance arrangements between the government and an Indigenous community controlled health service [20-25]. Three interventions were implemented in Indigenous community controlled health services [28,30,32] and the same number within government services [27,29,31]. One intervention was implemented in a primary care service that had a shared governance arrangement between an Indigenous community board and a private practice group [26]. Indigenous peoples desire to have control over their own health has been expressed through the establishment of community-governed or controlled health services. Due to the diversity of where Indigenous people live and their health and well-being needs, it is likely that a range of governance arrangements within countries would be ideal [39]. There are multiple governance structures [40] and three of those present in this review. Four of the interventions in this study that occurred within primary care services with a shared governance structure achieved an improved health outcome measure. All three interventions within a community controlled setting did not achieve an improved health outcome measure of interest to this review.

### Limitations

Whilst we believed this study question could have provided insight on important system or service level attributes of primary health care that lead to improved related health outcomes, the limitations of this study require these analyses to be interpreted with caution. Differences in health care policies relating to Indigenous peoples health and well-being and to the provision of primary health care vary between the countries of interest [41,42]. Even to the extent of within country differences, such as in Canada, where First Nations people have more access to enhanced program funding from Health Canada compared to those living in the territories [5]. These policies would fundamentally impact on access to primary care services differently for each population group, that could lead to better or poorer health outcomes measured in this study.

Vast differences in the setting, the population, the target group and the outcome measures limited the comparative value of the interventions. It is also not possible to attribute a change in health outcome solely to the intervention as external factors may have contributed and likely to a greater extent without participant randomisation or analysis that adjusts for confounding.

The study inclusion criteria deliberately sought an objective health outcome measure in determining the scope of the review. This excluded studies that reported subjective outcomes, which may have been otherwise well designed, omitting their contribution to the synthesised findings of this review. In addition, the number of eligible studies may have been limited by excluding gray literature in search strategy.

## Conclusions

A small number of published studies that measure the impact of diabetes management in a PHC setting on health outcomes among the four selected Indigenous population groups were found. Across these studies there were no consistent patterns of health system or service level attributes. However, the results do provide insight on the characteristics, quality and impact on health outcomes of the interventions being implemented in the four countries to address the escalating prevalence of type 2 diabetes in their Indigenous populations. In the last 15 years, Australia has predominantly invested in establishing and improving the ability of their clinical systems to manage chronic diseases including T2DM. Since 2010, efforts in NZ have involved increasing Maori peoples access to mainstream services. Canada’s response has been to provide service programs and build the capacity of the clinic system in hard to reach locations. The US has spread its efforts across building both the PHC system and the clinical system. All interventions in this review that had a positive impact on health outcomes supported the development of multiple areas, as opposed to having a single focus.

Descriptive/observational studies are the largest contributors to the evidence, possibly because they are more ethically acceptable to the participating communities and less time and resource intensive than comparable cohort studies and randomised trials. Failure to describe participants who withdrew from the intervention was a weakness in the quality of the observational studies. Characteristics of participants who withdrew from the intervention are a valuable finding that can help inform the appropriateness of future interventions. Mixed methods research that imparts perspectives of policy makers and funders, service providers and patients in addition to objective health outcomes could provide a more comprehensive understanding of how PHC attributes may impact on health outcomes. Conducting rigorous PHC research in real settings, especially at the health or clinical system level is difficult. However, this is an important question for policy makers, and further high quality research is needed to contribute to the evidence base for informed decision making.
